# The pangenome of an agronomically important crop plant *Brassica oleracea*

**DOI:** 10.1038/ncomms13390

**Published:** 2016-11-11

**Authors:** Agnieszka A. Golicz, Philipp E. Bayer, Guy C. Barker, Patrick P. Edger, HyeRan Kim, Paula A. Martinez, Chon Kit Kenneth Chan, Anita Severn-Ellis, W. Richard McCombie, Isobel A. P. Parkin, Andrew H. Paterson, J. Chris Pires, Andrew G. Sharpe, Haibao Tang, Graham R. Teakle, Christopher D. Town, Jacqueline Batley, David Edwards

**Affiliations:** 1 School of Agriculture and Food Sciences, The University of Queensland, Brisbane, Queensland 4072, Australia; 2 School of Plant Biology, The University of Western Australia, 35 Stirling Highway, Crawley, Western Australia 6009, Australia; 3 School of Life Sciences, The University of Warwick, Warwick CV35 9EF, UK; 4 Department of Horticulture, Michigan State University, East Lansing, Michigan 48823, USA; 5 Korea Research Institute of Bioscience & Biotechnology, 125 Gwahak-ro, Yuseong-gu, Daejeon 34141, Korea; 6 Cold Spring Harbor Laboratory, 1 Bungtown Road, Cold Spring Harbor, New York 11724, USA; 7 Agriculture and Agri-Food Canada, 107 Science Place, Saskatoon, Saskatchewan, Canada S7N0X2; 8 Plant Genome Mapping Laboratory, University of Georgia, Athens, Georgia 30602, USA; 9 Division of Biological Sciences, Bond Life Sciences Center, University of Missouri, Columbia, Missouri 65211-7310, USA; 10 National Research Council Canada, 110 Gymnasium Place, Saskatoon, Saskatchewan, Canada S7N0W9; 11 Center for Genomics and Biotechnology, Fujian Provincial Key Laboratory of Haixia Applied Plant Systems Biology, Haixia Institute of Science and Technology (HIST), Fujian Agriculture and Forestry University, Fuzhou, Fujian 350002, China; 12 J. Craig Venter Institute, 9704 Medical Center Drive, Rockville, Maryland 20850, USA

## Abstract

There is an increasing awareness that as a result of structural variation, a reference sequence representing a genome of a single individual is unable to capture all of the gene repertoire found in the species. A large number of genes affected by presence/absence and copy number variation suggest that it may contribute to phenotypic and agronomic trait diversity. Here we show by analysis of the *Brassica oleracea* pangenome that nearly 20% of genes are affected by presence/absence variation. Several genes displaying presence/absence variation are annotated with functions related to major agronomic traits, including disease resistance, flowering time, glucosinolate metabolism and vitamin biosynthesis.

## Abstract (web summary)

*Brassica oleracea* is a single species that includes diverse crops such as cabbage, broccoli and Brussels sprouts. Here, the authors identify genes not captured in existing *B. oleracea* reference genomes by the assembly of a pangenome and show variations in gene content that may be related to important agronomic traits

## Graphical abstract

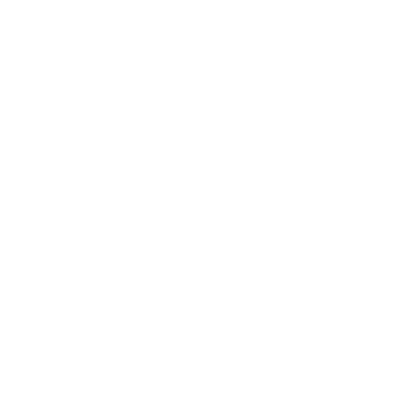

B*rassica oleracea* is a diploid, agronomically important plant species encompassing many popular crops, including cabbage, cauliflower, broccoli, Brussels sprout, kohlrabi and kale. *Brassica* crops display remarkable morphological diversity, and are grown for their inflorescences, axillary buds, leaves and stems. While two reference genomes of *B. oleracea* are available^1^,^2^, a reference sequence cannot capture the entire gene content of a species owing to structural variants, namely the presence/absence variants (PAVs) and copy number variants^3^,^4^,^5^,^6^. Plant reference genomes have been shown to lack a number of agronomically important genes, restricting the gene space available for analysis, for example, in association studies^7^. To address this, pangenomes have been constructed for a number of species, including maize, rice and soybean^7^,^8^,^9^. The term pangenome was first introduced by Tettelin *et al*.^10^ in 2005 and refers to a full genomic (genic) makeup of a species. Construction of a pangenome allows capturing of sequence affected by structural variation and possibly absent from the reference sequence of a single individual. A number of pangenome assembly approaches exist, including comparison of full *de novo* genome assemblies and reference guided assembly approaches^6^.

Here we describe the construction and analysis of a *B. oleracea* pangenome using nine morphologically diverse *B. oleracea* varieties and a wild relative—*Brassica macrocarpa*. The pangenome comprises 61,379 genes, 18.7% of which demonstrate PAV in the varieties analysed. Several of the variable genes are annotated with functions related to major agronomic traits, including disease resistance, flowering time, glucosinolate metabolism and vitamin biosynthesis, suggesting that PAVs may be important for the breeding of improved *Brassica* crops.

## Results

### Pangenome construction

**Figure 1:**
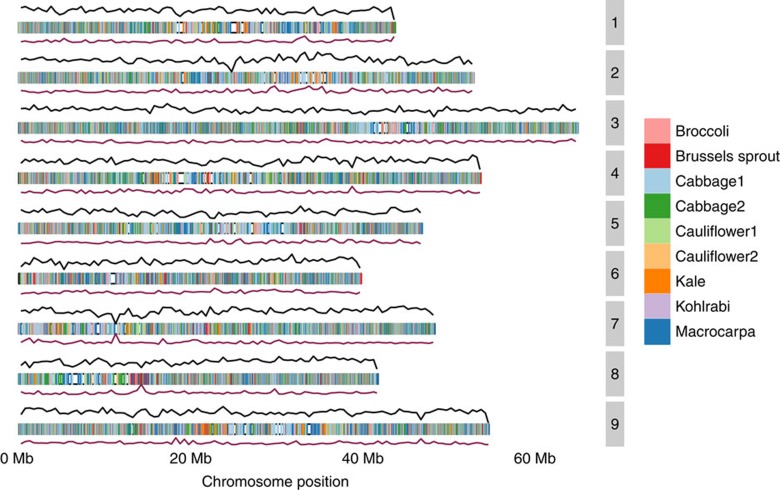
*B. oleracea* pangenome. SNP density, pangenome contig placement on TO1000 chromosomes and variable gene density. Each chromosome is represented by three tracks, which from the top correspond to the following: (1) SNP density (black line)—each chromosome was split into 500 kb bins, the number of SNPs in bins is plotted as a function of bin position; (2) pangenome contig placement on TO1000 chromosomes (coloured rectangle)—contigs originating from each step of pangenome construction were placed along the chromosomes and colour coded according to the line; (3) variable gene density (burgundy line)—the number of variable genes in each 500 kb bin is divided by the total number of genes.

The *Brassica* C pangenome was built using an iterative mapping and assembly approach, anchored by the publicly available genome of rapid cycling line TO1000 (ref. 2) and including additional sequences from nine other lines (eight cultivated lines and one wild type—*B. macrocarpa*, Supplementary Tables 1 and 2). The assembled pangenome is 587 Mbp in size and contains 61,379 gene models, compared with the *B. oleracea* var TO1000 assembly of 488 Mbp and 59,225 gene models (including 54,457 confident non-TE (transposable element) gene models used in the analysis; Supplementary Table 3 and Supplementary Fig. 1); and the 535 Mbp assembly and 45,758 gene models reported for *B. oleracea* var capitata (cabbage)^1^,^2^. Among the contigs contributed by nine additional lines, 28% could be placed along the nine TO1000 chromosomes using paired read sequence information (Fig. 1 and Supplementary Fig. 2).

### Gene presence/absence discovery and characterization

**Figure 2:**
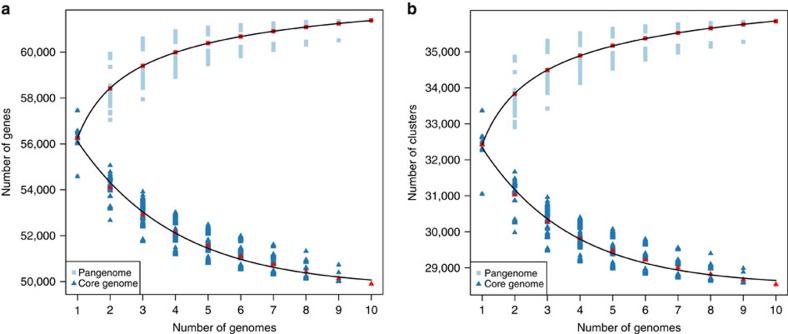
Model describing the sizes of core and pangenome. Every genome added using (**a**) all genes and (**b**) orthologous gene clusters. Red points correspond to mean value. The pangenome size increases with each added line up to 61,379 genes (35,853 gene families), and extrapolation of pangenome size leads to a predicted pangenome of 63,865±31 genes (37,766±62 gene families). The size of the core genome diminishes with every added line to 49,895 genes (28,532 gene families) with a predicted core genome size of 49,740±164 genes (28,496±91 gene families). ±corresponds to s.e.

**Figure 3:**
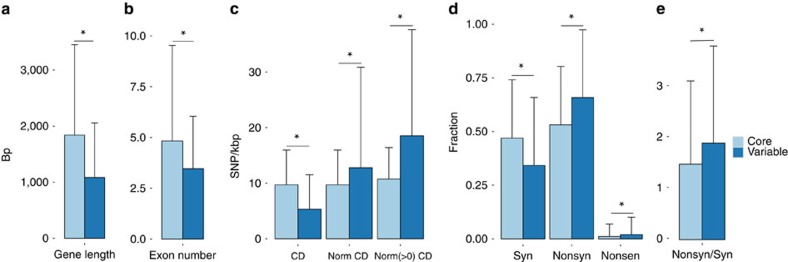
Comparison of core and variable genomes. Core and variable gene properties, SNP density and difference between core and variable genes with respect to synonymous SNPs, nonsynonymous SNPs and nonsense SNPs. Variable genes are on average (**a**) shorter with (**b**) fewer exons and (**c**) lower SNP density. After correction for the number of instances of a gene, coding mean SNP density (Norm CD) of variable genes is higher than core genes, but the ranks of core genes are higher (*U*-test). When genes with at least one coding SNP are considered (Norm(>0) CD), variable genes also have higher mean SNP density and the ranks of variable genes are higher (*U*-test). (**d**) There is a difference in the number of SNPs between core and variable genes within all three groups Syn (synonymous), Nonsyn (nonsynonymous) and Nonsen (nonsense). (**e**) Nonsyn/Syn ratio for core and variable genes. Bars correspond to means. Error bars correspond to s.d. *P<0.001, *U*-test. The total number of genes considered *n*=61,379 (core, *n*=49,895; variable, *n*=11,484).

The majority (81.3%, 49,895) of the pangenome is composed of core genes present in all lines, while 18.7% (11,484) of the genes are variable, with 2.2% (1,322) present in one line only (Supplementary Fig. 3). Modelling of pangenome size (Fig. 2) suggests a closed (restricted) pangenome with a finite number of genes (orthologous gene clusters), consistent with pangenome analyses in maize^8^ and soybean^9^. Variable genes were shorter than core genes, with fewer exons per gene (Fig. 3a,b and Supplementary Table 4), consistent with previous reports concerning genes displaying PAV^11^,^12^.

TE density surrounding core and variable genes was investigated. Higher TE density surrounding variable genes (compared with the core genes) was observed (*U*-test, *P*<0.001). In addition, a higher proportion of haT superfamily transposons and long interspersed nuclear elements in the vicinity of variable genes was observed (Supplementary Fig. 4). Long interspersed nuclear elements have previously been found to be associated with structural variants, and are thought to mediate structural variant generation via non-allelic homologous recombination^13^,^14^. Similarly, it was suggested that haT superfamily transposons mediate structural variant formation via alternative transposition^15^.

In total, 4,815,081 single-nucleotide polymorphisms (SNPs) were identified in the pangenome with an overall SNP density of 8.2 SNPs per kb (Supplementary Table 5). Private SNP abundance varied between *B. macrocarpa* and Cauliflower1 (Supplementary Fig. 5). There was greater SNP density within the coding regions of core genes than variable genes. However, when SNP density was adjusted for the number of instances of a gene, the variable genes had higher SNP density (Fig. 3c). Core genes have a greater proportion of synonymous SNPs and a lower proportion of nonsynonymous and nonsense SNPs than variable genes (Fig. 3d,e).

**Figure 4:**
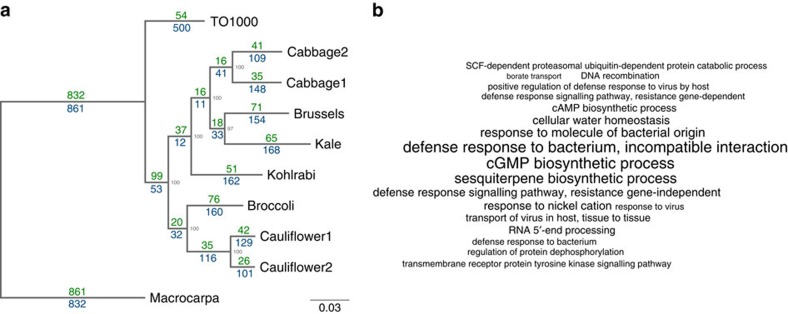
Phylogeny and gene ontology enrichment (**a**) Phylogenetic tree presenting relationships between the 10 varieties. The number of presence/absence genes were placed on the phylogenetic tree, so that the branch leading to a node was labelled with the genes uniquely present and absent in all lines below the node. Genes absent are shown in blue and genes present in green. Scale bar indicates number of nucleotide substitutions per site. (**b**) Significantly enriched gene ontology terms among variable genes using all pangenome genes as a background. Font size is proportional to –log(*p*).

A phylogenetic tree of relationships between the 10 *Brassica* genotypes was built using RAxML (Fig. 4a). Overall, 4,324 (37.7%) gene PAVs were consistent with the phylogenetic estimates of relationships and may represent morphotype-lineage-specific gene PAV. The largest number of uniquely present and absent genes was found in *B. macrocarpa*, which reflects its greater evolutionary distance from the other samples^16^, while the line with the second greatest number of uniquely absent genes was the TO1000 rapid cycling line.

### Functional analysis of variable genes

Functional analysis of variable genes suggests enrichment of genes and gene families involved in disease resistance, defence response, water homeostasis, amino-acid phosphorylation and signal transduction (Fig. 4b, and Supplementary Tables 6 and 7). PAV among defence response (biotic stress) genes has been observed in several plant species^17^,^18^. The presence/absence of resistance genes could partially be due to their overlapping roles and large number available for deletion following a whole-genome triplication event shared by the *Brassica* species^1^,^19^,^20^, however the presence of pathogens is also likely to impact gene retention due to strong selection for corresponding resistance genes. In total, 439 putative resistance genes were identified, including 251 core and 188 variable genes (Supplementary Fig. 6). The genes were classified in different categories based on presence of leucine-rich repeat (LRR), toll/interleukin-1 receptor-like (TIR) and coiled-coil (CC) domains (Supplementary Table 8). The genes were distributed unevenly across chromosomes, which is similar to observations made in other plants^21^,^22^, and an estimated 45% of nucleotide binding site (NBS) domain-containing genes were found in clusters.

Functional annotation of morphotype-lineage-specific PAV highlights genes involved in biotic and abiotic stress responses. These may reflect the evolution or breeding for adaptive traits. *B. macrocarpa*, which possesses a large number of uniquely present genes, has previously been identified as a potential donor of valuable traits^23^. Functional analysis suggests presence of unique genes involved in defence response, response to salt stress, cold and water deprivation (Supplementary Table 9).

### Presence/absence variation of auxin-related genes

Whole-genome triplication contributed to expansion of gene families involved in auxin functioning (*AUX*, *IAA*, *GH3*, *PIN*, *SAUR*, *TIR*, *TPL* and *YUCCA*), and morphology specification (*TCP*), and duplicated genes may contribute to the extraordinary morphological variation in *Brassica* species^1^. As PAV among those genes may also be a contributing factor, the homologues of auxin-related genes and *TCP* were assessed. PAV within auxin-related genes but not *TCP* was detected (Supplementary Table 10).

### Presence/absence variation of flowering related genes

**Figure 5:**
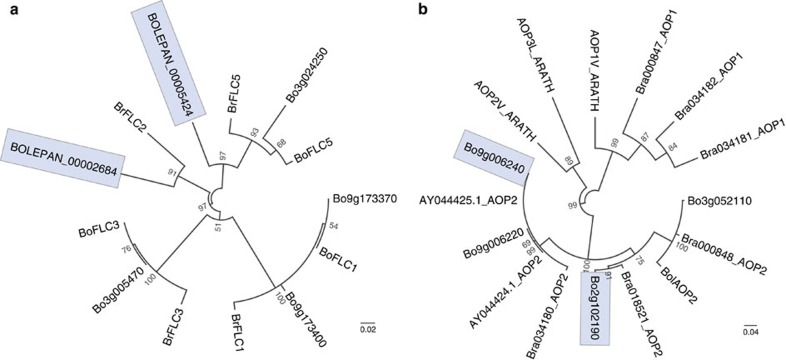
Phylogenetic trees presenting relationships between *FLC* and *AOP2* gene protein products. (**a**) There are six *FLC* genes identified in the pangenome. BrFLC1, 2, 3, 5 and BoFLC1, 3, 5 are protein products of *B. rapa* and *B. oleracea FLC* genes, respectively, previously identified by Schranz *et al*.^27^. Bo3g005470, Bo3g024250, Bo9g173370 and Bo9g173400 are protein products of genes identified on the TO1000 portion, and BOLEPAN_00002684 and BOLEPAN_00005424 on the newly assembled portion of the pangenome. (**b**) There are four *AOP2* genes identified in the pangenome. AOP1V_ARATH, AOP2V_ARATH and AOP3L_ARATH are protein products of *AOP1, 2, 3* genes identified in *A. thaliana*. BolAOP2 is a protein product of the only full-length *AOP2* gene identified in *B. oleracea* var capitata genome^1^. Proteins with IDs beginning with Bra are products of *AOP* genes identified in *B. rapa* and proteins with IDs beginning with Bo are products of *AOP* genes identified in the *B. oleracea* pangenome in this study. Genes *Bo9g006220* and *Bo9g006240* co-localize with quantitative trait locus (QTL) controlling amount of several glucosinolates^70^. Genes displaying PAV are highlighted in blue rectangles. Scale bars indicate number of amino-acid substitutions per site.

*B. oleracea* grows in a range of climatic zones and latitudes, and different cultivars have been selected for flowering time and maturity. There were 14 variable genes predicted to be involved in flowering, with TO1000 demonstrating the greatest absence of flowering genes (Supplementary Table 11 and Supplementary Fig. 7). A similar observation was made in *Brassica rapa*, where a rapid cycler was also missing several flowering time-related genes^24^, suggesting that PAV may be a contributing factor of flowering time regulation. The genes identified include orthologues of genes encoding: *MAF5*, *SEP2*, *ARP4*, *GID1B*, *FPF1*-like, *FHY1*, *GA2*, *GA3* and *CO*. Flowering locus C (*FLC*) is an important regulator of vernalization and flowering time. *FLC* is thought to control flowering in a dosage-dependent manner, and the flowering time variation appears to be affected by the number of copies of *FLC* gene present^25^. Only one *FLC* gene is present in *Arabidopsis*, whereas four, four and five genes have been identified in *B. rapa*, *B. oleracea* and *B. napus*, respectively^26^,^27^,^28^ A recent whole-genome assembly of *B. napus* allowed identification of nine *FLC* genes (four on the A genome and five on the C genome) and identified four *FLC* paralogues in *B. oleracea* var TO1000 (ref. 21). These four were identified in all lines examined, and two additional candidate *FLC* genes were discovered; one (*BoFLC2*) present in all lines except TO1000 and the other (*BoFLC5*, partial gene model) present only in *B. macrocarpa* and Cauliflower1 (Fig. 5a). Studies in *B. rapa* have shown *BrFLC2* to be a key regulator of flowering time^29^,^30^,^31^. In cauliflower, the disrupted *BoFLC2* allele was associated with early flowering^32^. In addition, previous studies noted lack of hybridization of a *BoFLC2* probe in a rapid cycling variety suggesting existence of an underlying deletion or substantial sequence variation^27^,^28^. The PAV analysis presented together with comparison of the contig harbouring the *BoFLC2* gene with the TO1000 genome suggest that a deletion is a likely cause of *BoFLC2* absence. Furthermore, it is a likely contributor to the early flowering phenotype of rapid cycler, TO1000.

### Presence/absence variation of glucosinolate-related genes

The number of variable genes involved in biosynthesis and breakdown of secondary metabolites (glucosinolates, carotenoids, ascorbate, tocopherol and anthocyanin) was assessed (Supplementary Tables 12 and 13, and Supplementary Fig. 8). In total, eight variable genes involved in glucosinolate biosynthesis and breakdown were observed. The variable genes included orthologues of genes involved in core structure formation, cytochrome *CYP79A2*, *SUR1* and *SOT18*, and side chain modification (*AOP2*). Comparison of glucosinolates between Brassicas suggests significant variation in glucosinolate types and abundance^33^. Our analysis suggests that gene PAV may be a contributing factor to the diversity observed. Previous reports in *Arabidopsis* and other *Brassica* relatives have shown that gene duplications and subsequent sequence divergence contribute to glucosinolate pathway diversification^21^,^34^,^35^,^36^. AOP2 catalyses the conversion of methylsulphinylalkyl glucosinolates to corresponding alkenyl glucosinolates. It is of particular interest in *Brassica* crops because it catalyses conversion of health protective glucoraphanin, into deleterious products. In *B. rapa*, three functional, differentially expressed *AOP2* genes were observed^37^. Broccoli harbours a non-functional *AOP2* allele and accumulates glucoraphanin^38^. In cabbage, one full-length and two truncated *AOP2* proteins were reported^1^. In TO1000, four *AOP2* genes were identified, two of these display PAV (absent in *B. macrocarpa*) (Fig. 5b). Two of the genes involved in ascorbate biosynthesis were variable (orthologues of L-galactose dehydrogenase), however none of the genes involved in carotenoid, tocopherol and anthocyanin biosynthesis were variable.

## Discussion

The observation that nearly 18.7% of the pangenome is composed of variable genes may have implications for breeding. It is commonly recognized that different *Brassica* crop types have a restricted set of alleles compared with the wider species genepool, and here we show that some of these variations can be attributed to PAV. Performing wider crosses between crop types will give access to additional genes not present in a particular *Brassica* crop type. In addition, PAV may also contribute to the phenomenon of heterosis in F1 hybrids, since the presence of additional genes, even in heterozygous state, may give rise to increased vigour^6^,^39^,^40^. Finally, the finding that *B. macrocarpa* possesses the largest number of uniquely present genes suggests that *Brassica* wild relatives represent a significant source of new genes that were lost during domestication.

## Methods

### Pangenome assembly

Sequence data are listed in Supplementary Table 1. The pangenome was assembled using an iterative mapping and assembly approach. The approach is related to previously described reference-guided approaches^7^,^8^,^41^. The iterative mapping and assembly strategy was chosen considering the nature of the data. Lack of long insert libraries resulted in highly fragmented whole-genome assemblies, which made whole-genome alignment challenging^6^. The publicly available reference sequence for a Chinese kale rapid cycling line (TO1000)^2^ was used as a reference for pangenome construction. The procedure involved three main steps: mapping of the reads to the reference sequence; assembly of the unmapped reads; and production of a new reference sequence by updating the old one with the newly assembled contigs. The mappings and assembly were performed in the following order: Cabbage1; Cabbage2; Kale; Brussels sprout; Kohlrabi; Cauliflower1; Cauliflower2; Broccoli; and Macrocarpa. Different orders were tested however the resulting assembly sizes were similar regardless of order used (Supplementary Fig. 9). Mapping was performed using Bowtie2 (ref. 42) v2.2.5 (--end-to-end --sensitive -I 0 -X 1000) and assembly was performed using MaSuRCA^43^ v3.1.3. The TO1000 genome and the newly assembled contigs together constituted the pangenome. Mitochondrial (NC_016118.1) and chloroplast (NC_015139.1) genomes were included in the mappings (added to the TO1000 genome sequence) to eliminate potential plastid contamination. Before assembly, adapters were removed using Trimmomatic^44^ v0.36. The assembly was validated by remapping the reads to the assembly (Supplementary Figs 10–12)

### Sequencing

For all samples, DNA was extracted from leaf tissue. For CA25, AC498, ARS_18 and HRIGRU011183, DNA was extracted using the megabase-sized isolation protocol^2^. Illumina paired-end libraries with 300–500 bp insert size were prepared following the manufacturer's instructions and sequenced using HiSeq2000 (100 and 101 bp reads) and HiSeq2500 (126 bp reads). For Badger Inbred 16, HRIGRU009617, BOL909 and *B. macrocarpa*, DNA was extracted using the QIAGEN DNeasy plant mini kit. Illumina paired-end libraries with 300–350 bp insert size were prepared following the manufacturer's instructions and sequenced using HiSeq2000. For Early Big, DNA was extracted using the CTAB procedure. Illumina paired-end library with 350 bp insert size was prepared following the manufacturer's instructions and sequenced using the Genome Analyzer II.

### Bacterial contamination

BLAST^45^ v2.2.30 against NCBI nt database (03.05.2016) was used to identify and remove potential contamination. Contigs whose best hits were not against green plants were tagged as contamination. Contaminant contigs were included in all the mappings but not included in the subsequent analysis.

### Alignment of the newly assembled contigs to the pangenome

The newly assembled contigs from each stage of the assembly were aligned to the portion of the pangenome, which served as a reference at this stage using LASTZ v1.02.00 (--notransition --chain --ambiguous=n --identity=93 --coverage=90 --continuity=95). The sequence identity cutoff was estimated based on *B. rapa* and *B. oleracea* divergence. *B. rapa* and *B. oleracea* chromosomes 1 were aligned using LASTZ v1.02.00 (--chain --ambiguous=n, genic regions were masked on both chromosomes). The per cent identity cutoff value was calculated as follows (estimated *B. oleracea* wild species divergence time) × (first quartile *B. rapa*−*B. oleracea* per cent identity)/(estimated *B. rapa*−*B. oleracea* divergence time). The *B. oleracea* wild species divergence time used was 1.44 myr ago (ref. 46). The *B. rapa*−*B. oleracea* divergence time used was 2.54 myr ago (ref. 46).

### Pangenome annotation

Newly assembled contigs ≥1,000 bp in length were annotated using MAKER2 (ref. 47) v2.31.8. *De novo* gene prediction used SNAP^48^ and Augustus^49^, the EST evidence was based on *B. oleracea* genes downloaded from UniGene (ftp://ftp.ncbi.nlm.nih.gov/repository/UniGene/Brassica_oleracea/Bol.seq.uniq.gz) and 95 k ESTs (http://brassica.nbi.ac.uk/array_info.html), while protein evidence was based on Brassicaceae proteins downloaded from RefSeq. Publically available RNASeq data were downloaded from SRA and used as additional evidence. Sequence was masked against ‘te_proteins.fasta' in the MAKER2 package. The total number of genes predicted may be underestimated due to lack of comprehensive RNASeq libraries for all the lines used in the analysis.

TE-related genes were detected using hmmsearch^50^ v3.1b2 (trusted cutoff) using 137 identified TE-related domains^51^.

R genes were identified using hmmsearch v3.1b2 (trusted cutoff). Genes that contained PF00931 (NB-ARC) domain were considered to be R genes. The LRR and TIR domains were also assigned based on hmmsearch results. The CC domain was discovered using Paircoil2 (ref. 52). Resistance gene clusters were determined by their physical position order^22^,^53^,^54^. The parameter to define a cluster was two or more R genes that occurred within a maximum of ten non-R and R genes. The neighbour joining tree was drawn using QuickTree^55^ v1.1.

### Placement of contigs along TO1000 chromosomes

The newly assembled contigs >200 bp in length were placed along the TO1000 chromosomes using paired-end read information. Reads from the nine varieties (Cabbage1, Cabbage2, Kale, Brussels sprout, Kohlrabi, Cauliflower1, Cauliflower2, Broccoli and Macrocarpa) were mapped to the pangenome (TO1000 genome and the newly assembled contigs) using Bowtie2 v2.2.5 (--end-to-end --sensitive -I 0 -X 1000). Duplicates were marked using Picard tools MarkDuplicates. Each of the mappings were processed separately looking only at contigs that originated from a given line. Reads mapping to the first and last 300 bp of each newly assembled contig, which had mates mapping to a different chromosome/contig, were extracted using Samtools^56^. Only reads fulfilling the following criteria were extracted: MAPQ ≥10 (−q 10); not mapped in proper pairs (−F 2); not unmapped (−F 4); mate not unmapped (−F 8); and not duplicate (−F 1024). All the extracted reads were inspected for the mapping position of the mate. If mates of 80% or more of the inspected reads from one end of a contig mapped to a single chromosome and the mapping positions of mates (leftmost mapping coordinate) did not span more than 1,000 bp, this end of the newly assembled contig was placed on the chromosome and assigned a position equal to the median of mates leftmost mapping coordinates. If both ends of a newly assembled contig were placed on a single chromosome, a lower placement coordinate was assigned. In case of conflicts no placement was made. Each placement had to be supported by at least 10 reads.

### Gene presence/absence variation discovery

Gene presence/absence variation was characterized using the SGSGeneLoss package^57^. Reads from the 10 lines were mapped to the pangenome using Bowtie2 v2.2.5 (--end-to-end --sensitive -I 0 -X 1000). Reads from lines were subsampled to ∼25 × using Seqtk v1.1. Only reads mapping in proper pairs were retained. SGSGeneLoss utilizes a depth-of-coverage calculation across all exons of the gene and calls gene absence when the horizontal coverage across exons (total number of exon bases covered by reads) of the gene was <5%. Only genes that were annotated on contigs with a length ≥1,000 bp were used in this analysis. A gene was considered core if it was present in all lines and variable if it was absent in at least one line.

### Presence/absence validation

Presence/absence gene calls were validated using PCR. Primers were designed for 28 genes (35 primers in total, Supplementary Tables 14 and 15). Presence/absence was tested in five varieties (Cabbage1, Brussels sprout, Cauliflower2, Kale and Kohlrabi).

### Gene clustering

Genes were clustered using OrthoMCL^58^ v2.09 (default parameters). *B. oleracea* pangenome genes were clustered with *A. thaliana* genes^59^ (TAIR 10), and gene families were divided into core and variable. A gene family was considered to be core if at least one gene in the family was present in all the varieties. The gene family was considered variable if the whole gene family was missing from at least one line.

### Pangenome modelling

Curves describing pangenome size, core genome size and new gene number for both individual genes and genes families were fitted in R using the nls function (nonlinear least squares) from package stats. Points used in regression corresponded to all the possible combinations of genomes. The combinations of genomes were obtained according to the following formula: 10!/(*n*!(10−*n*)!), *n*=[1,10], and the pangenome size was modelled using the power law regression *y*=*Ax*^*B*^+*C* (refs 10, 60). The core genome size was modelled using exponential regression *y*=*Ae*^*Bx*^+*C*. The model was fitted using means.

### TE annotation

TE elements were discovered using RepeatMasker^61^ using a *B. oleracea* repeat database. TE density surrounding genes was calculated as a proportion of base pairs annotated as TE in the 2,000 bp window preceding the start and following the end of gene.

### SNP discovery

Mappings used for contig placement were also used for SNP discovery. Duplicates were marked using Picard tools MarkDuplicates. SNPs were discovered using Platypus^62^ v0.7.9.1 (--minMapQual=30 --minBaseQual=20). The SNP discovery model was diploid. The samples used were doubled haploids or highly inbred. Although a low percentage of SNPs are expected to be heterozygous, the vast majority of the SNPs are expected to be homozygous. Heterozygous SNPs were considered potential artefacts and removed. SNPs were categorized as coding, synonymous, nonsynonymous and nonsense using R package VariantAnnotation^63^ v1.13.46.

### Comparison of core and variable genes

Core and variable genes were compared with respect to gene length, exon number, coding SNP density, synonymous (not resulting in amino-acid change), nonsynonymous (resulting in amino-acid change), nonsense (introducing premature stop codon) SNP numbers and nonsynonymous/synonymous SNP ratio. All the pangenome genes were split into two groups corresponding to core and variable genes. The data did not meet parametric test assumptions and the groups were compared using Mann–Whitney *U*-test as implemented in R function wilcox.test (two-tailed test). No assumptions about similarity of shapes of distributions were made.

### Phylogenetic analysis

Phylogenetic trees were constructed using RAxML^64^ v8.1.22 (-V -m ASC_GTRCAT --asc-corr=lewis -o Macrocarpa -p 12345 -# 20). Bootstrapping was performed using 100 replicates.

### Placement of genes on the phylogenetic tree

All the possible combinations of lines were analysed and for each combination lists of genes uniquely present and absent in these lines was obtained. For example combination Broccoli–Cauliflower1–Cauliflower2 will have two corresponding lists of genes: (1) genes found only in those three lines; the genes are present in all three lines, but absent in all the others; (2) genes absent only in those lines; the genes are absent in all three lines, but present in the others. The number of present and absent genes was then placed on the phylogenetic tree, so that the branch leading to a node was labelled with all the genes uniquely present and absent in all lines below the node.

### Gene ontology annotation

The pangenome was functionally annotated using Blast2GO (ref. 65) command line v2.5. All the pangenome genes were compared with *A. thaliana* proteins pre-formatted to comply with Blast2GO naming requirements (ftp://ftp.arabidopsis.org/Sequences/blast_datasets/other_datasets/CURRENT/At_GB_refseq_prot.gz). Comparisons were made using BLAST v2.2.30. Enrichment was performed using Fisher exact test as implemented in topGO^66^ package with method ‘elim' used to adjust for multiple comparisons.

### Detection of clusters enriched in variable genes

Clusters were constructed using OrthoMCL as described above. Clusters significantly enriched in variable genes were identified using Fisher exact test (*P* value <0.001). Functional annotation of clusters was performed by assigning functions of *A. thaliana* genes to the whole cluster.

### Annotation of phylogeny-specific variable genes

Annotation of phylogeny-specific variable genes was done by counting the abundance of gene ontology terms for genes assigned to each node/branch.

### Pathway annotation

The pathways involved in glucosinolate, carotenoid, ascorbate, tocopherol and anthocyanin biosynthesis and metabolism were identified from the *A. thaliana* metabolic pathway database (ftp://ftp.plantcyc.org/Pathways/Data_dumps/PMN9_September2014/aracyc_pathways.20140902, version downloaded on 24.02.2015). The pathways and corresponding genes were extracted. Genes associated with flowering time listed in ref. 24 were downloaded. All the genes belonging to the pathways of interest and the flowering time genes were compared with the orthologous gene clusters. *B. oleracea* genes associated with pathways/processes were identified as follows: if a cluster contained an *A. thaliana* gene belonging to the pathway all the *B. oleracea* genes belonging to this cluster were extracted and assigned to the pathway. Subsequently, it was determined if the *B. oleracea* gene's best *A. thaliana* BLAST hit is directly involved in the pathway, if that was the case, the *B. oleracea* gene was deemed to be involved. The four *B. oleracea FLC* paralogues were taken from Chalhoub *et al*.^21^

### Analysis of *FLC* and *AOP* genes

The *B. rapa* and *B. oleracea FLC* gene accessions were obtained from Schranz *et al*.^27^ The *B. rapa* and *B. oleracea AOP* genes were obtained from Liu *et al*. 1 and Wang *et al*.^67^ *A. thaliana AOP* proteins were obtained from Swiss-Prot. The sequences were aligned using Clustal Omega^68^ and a maximum likelihood tree with 500 bootstraps was constructed using MEGA6 (ref. 69).

### Data availability

The code used for presence/absence detection have been made available at http://www.appliedbioinformatics.com.au/index.php/SGSGeneLoss. All sequencing data that support the findings of this study have been deposited in the National Center for Biotechnology Information Sequence Read Archive and are accessible through the SRA accession numbers PRJNA301390, PRJNA248388 and SRR074124. Additional data used in the study are available at http://www.appliedbioinformatics.com.au/index.php/BOLPANGENOME. All other data supporting the findings of this study are available in the article and its Supplementary Information files or are available from the corresponding author on request.

## Additional information

**How to cite this article**: Golicz, A. A. *et al*. The pangenome of an agronomically important crop plant *Brassica oleracea*. *Nat. Commun.* **7**, 13390 doi: 10.1038/ncomms13390 (2016).

**Publisher's note:** Springer Nature remains neutral with regard to jurisdictional claims in published maps and institutional affiliations.

## Supplementary Material

Supplementary Information Supplementary Figures 1-12 and Supplementary Tables 1-15
