# Supplementary material for: The pangenome of an agronomically important crop plant Brassica oleracea
Source: Nat Commun. 2016 Nov 11;7:13390. doi: 10.1038/ncomms13390 (PMC5114598; doi:10.1038/ncomms13390)
Supplement: Supplementary Information — Supplementary Figures 1-12 and Supplementary Tables 1-15 [file ncomms13390-s1.pdf]

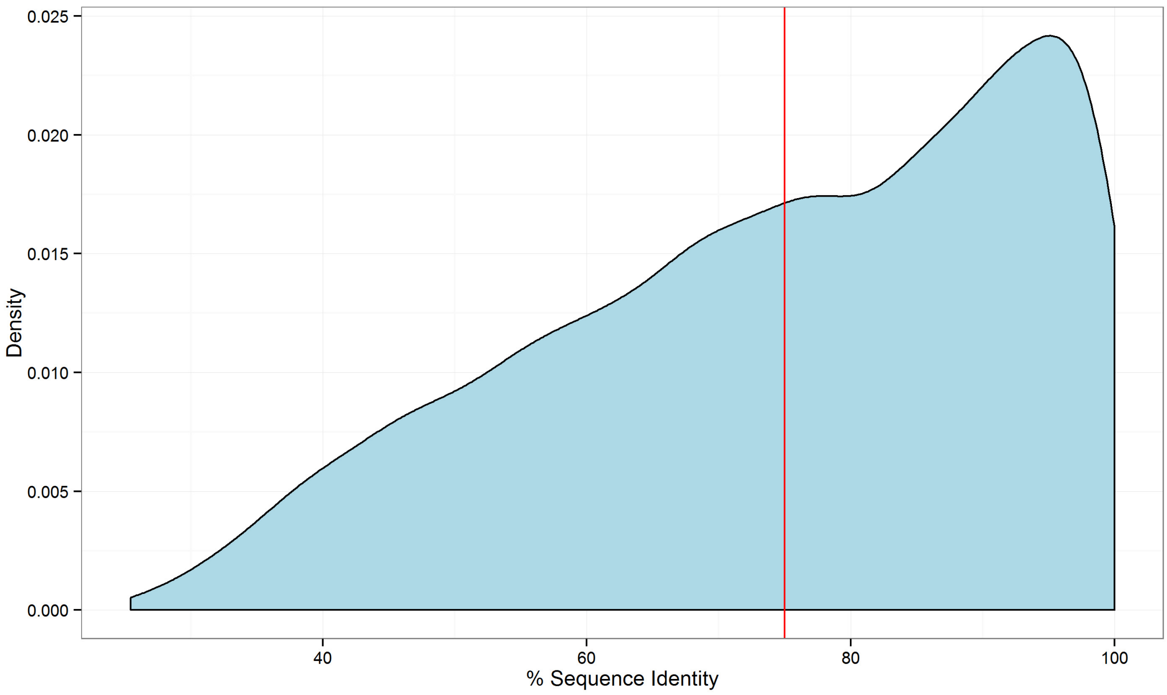

Supplementary Figure 1. Density plot of % identity (in protein space, as calculated by blastp) while comparing newly annotated proteins to the TO1000 proteome. Red vertical line corresponds to mean.

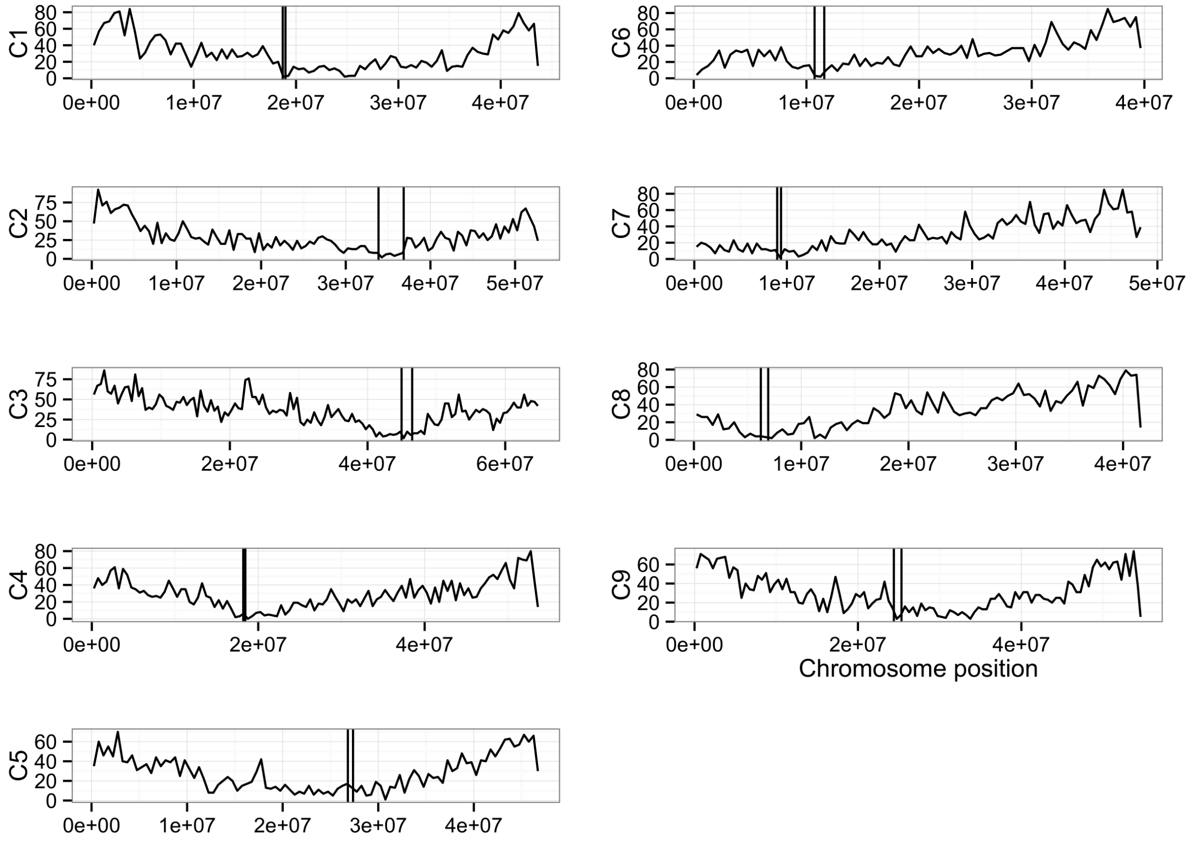

Supplementary Figure 2. Frequency of contig placements along the TO1000 chromosomes. Vertical lines delimit centromeres.

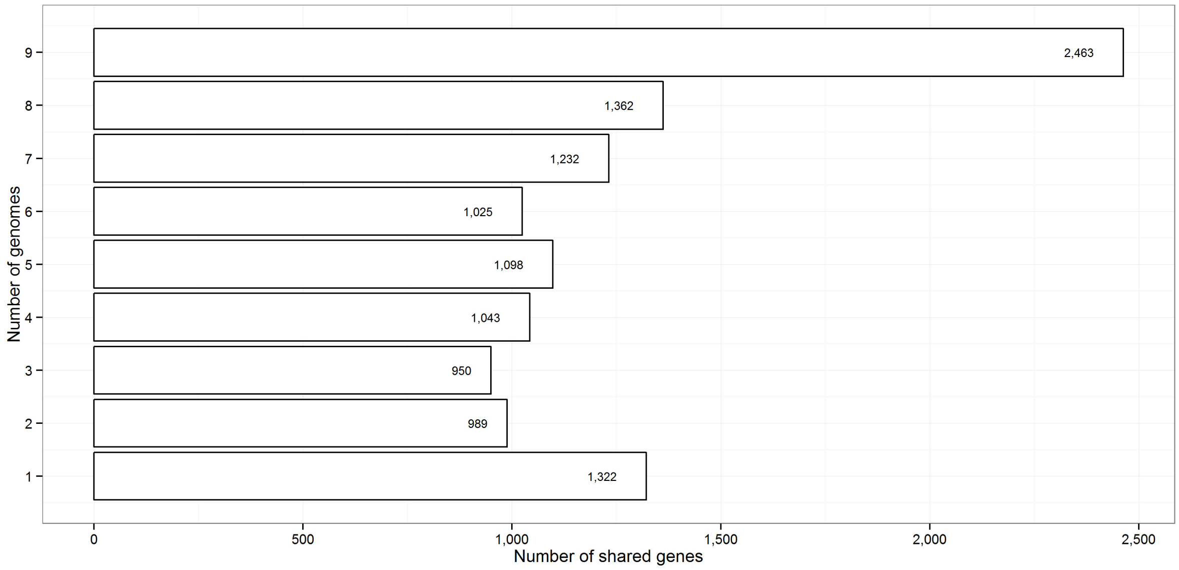

Supplementary Figure 3. Number of variable genes shared by different number of lines. For example, 950 genes were present in three lines, but absent in 7.

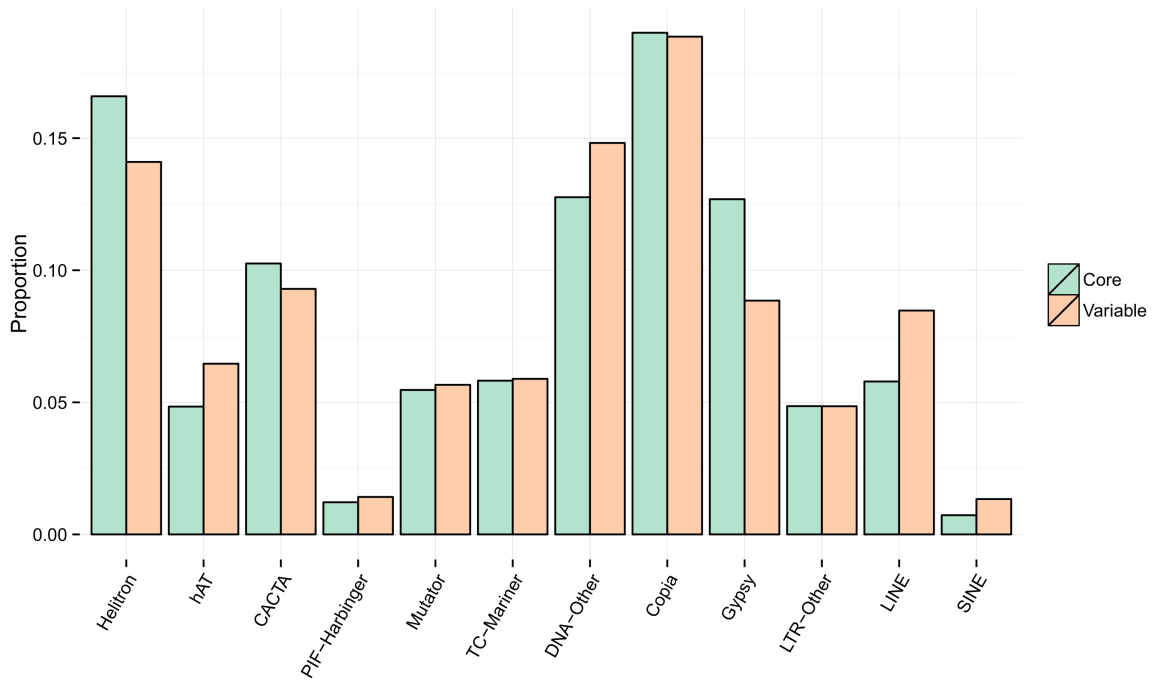

Supplementary Figure 4. Proportion of transposable element orders/superfamilies found in the vicinity (2000 bp window) of core and variable genes.

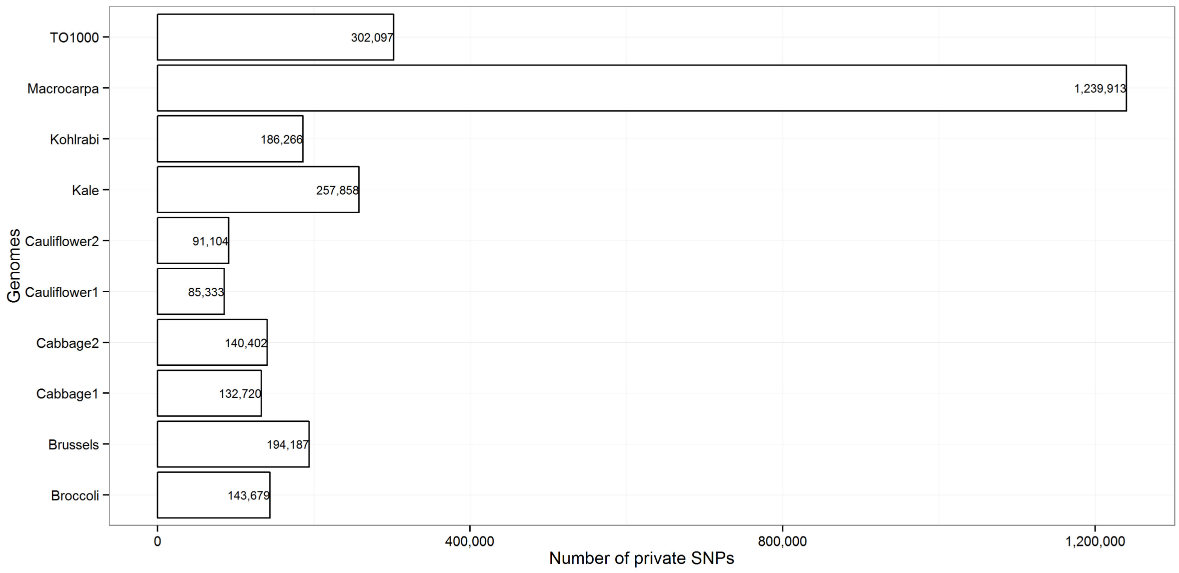

Supplementary Figure 5. Number of private SNPs present in each variety.

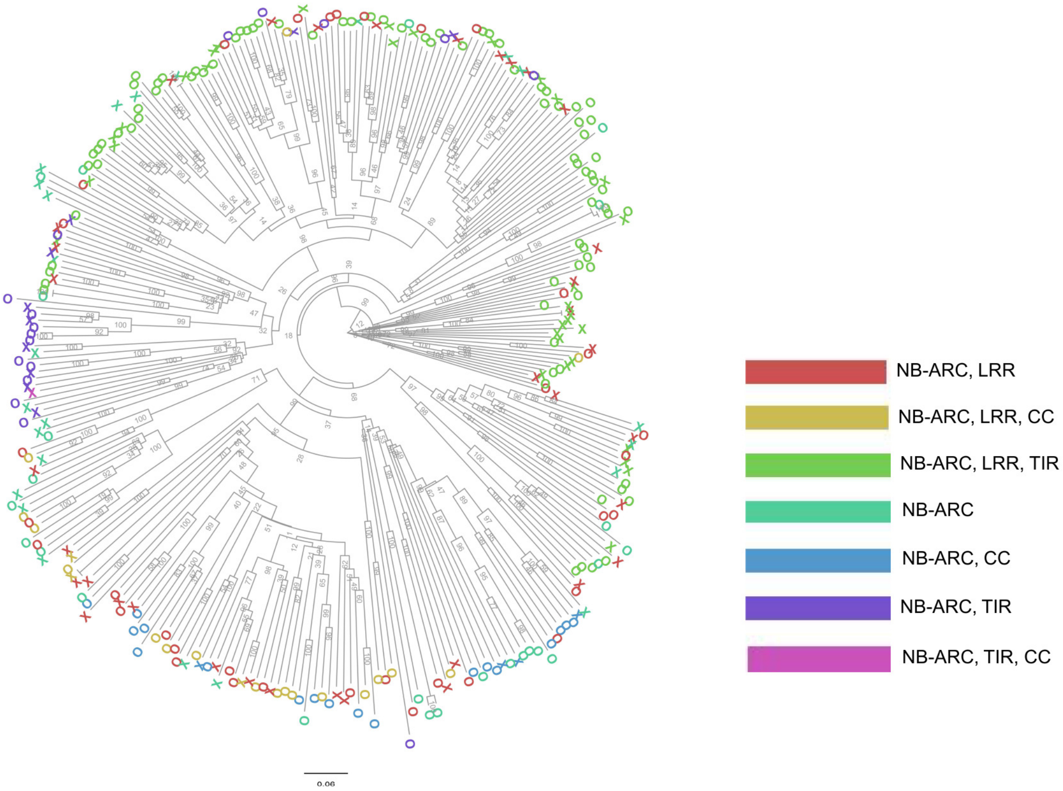

Supplementary Figure 6. Relationships between NB-ARC genes, X-variable gene, O-core gene. The NB-ARC domains were extracted, aligned using ClustalOmega (http://www.ebi.ac.uk/Tools/msa/clustalo/) and used to construct a neighbor joining tree showing relationships between NB-ARC genes. The neighbour joining tree was drawn using QuickTree.

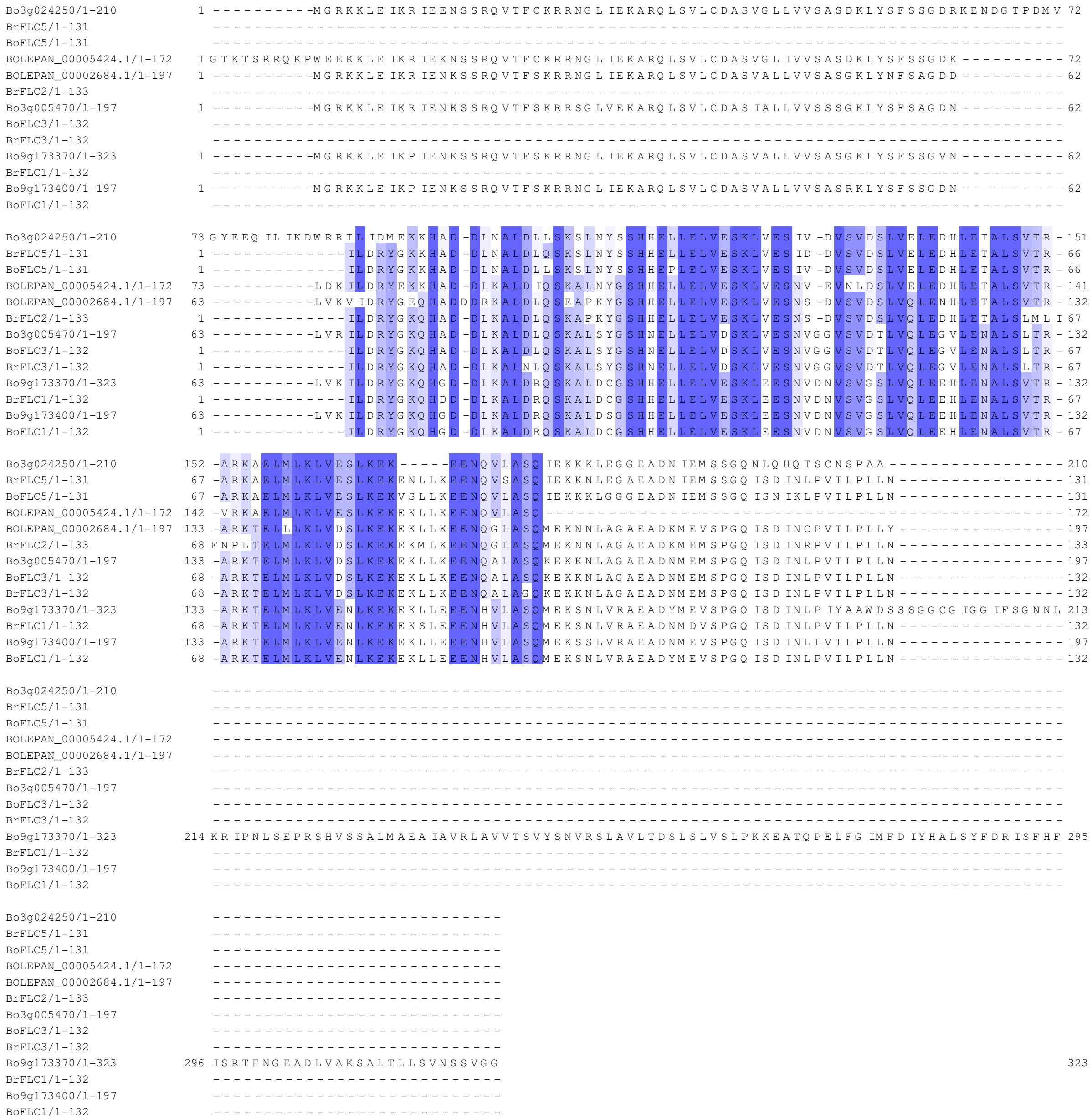

Supplementary Figure 7. Multiple sequence alignment of FLC proteins.

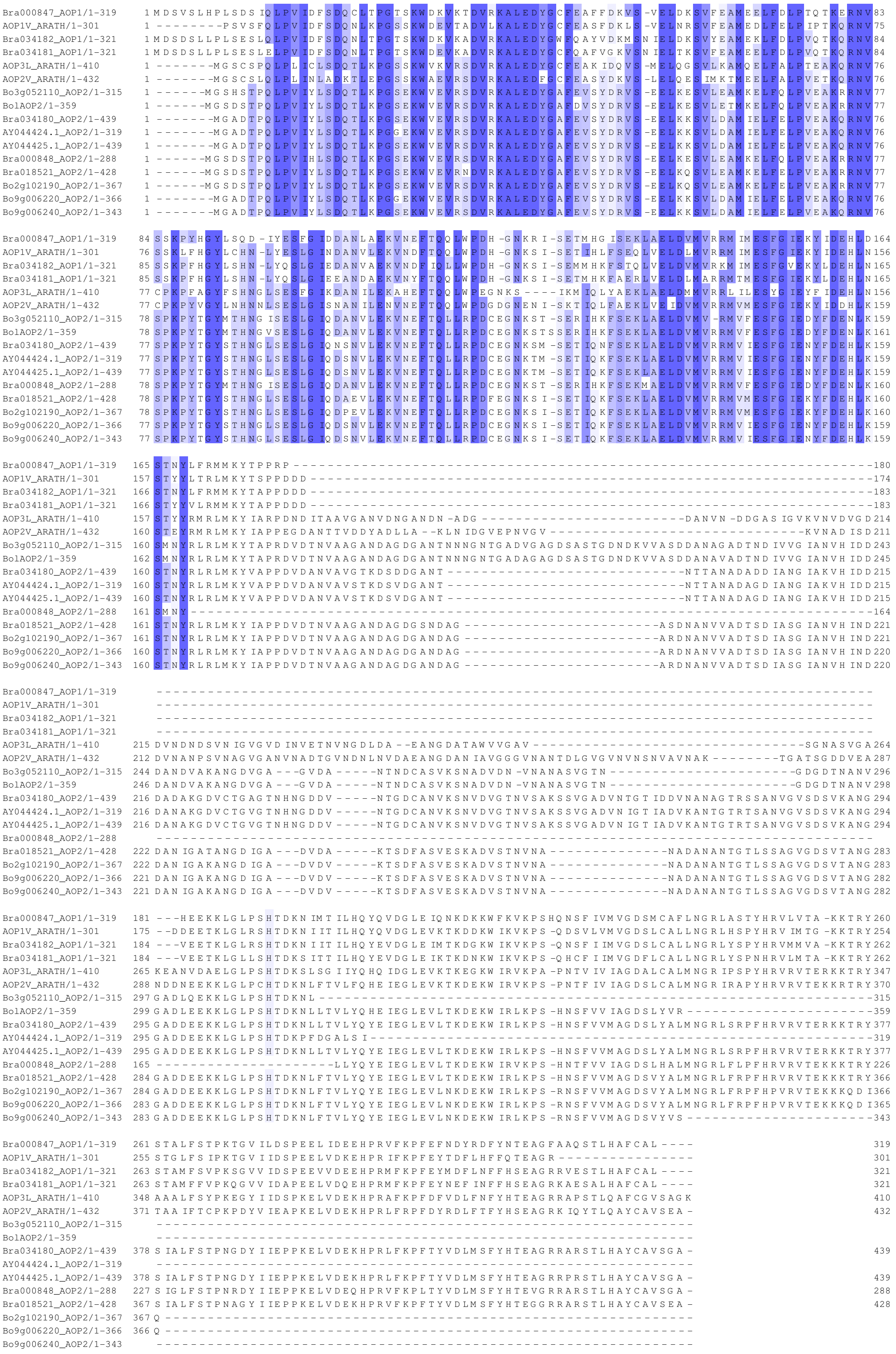
Bra000847_AOP1/1-319
AOP1V_ARATH/1-301
Bra034182_AOP1/1-321
Bra034181_AOP1/1-321
AOP3L_ARATH/1-410
AOP2V_ARATH/1-432
Bo3g052110_AOP2/1-315
BolAOP2/1-359
Bra034180_AOP2/1-439
AY044424.1_AOP2/1-319
AY044425.1_AOP2/1-439
Bra000848_AOP2/1-288
Bra018521_AOP2/1-428
Bo2g102190_AOP2/1-367
Bo9g006220_AOP2/1-366
Bo9g006240_AOP2/1-343

Supplementary Figure 8. Multiple sequence alignment of AOP proteins.

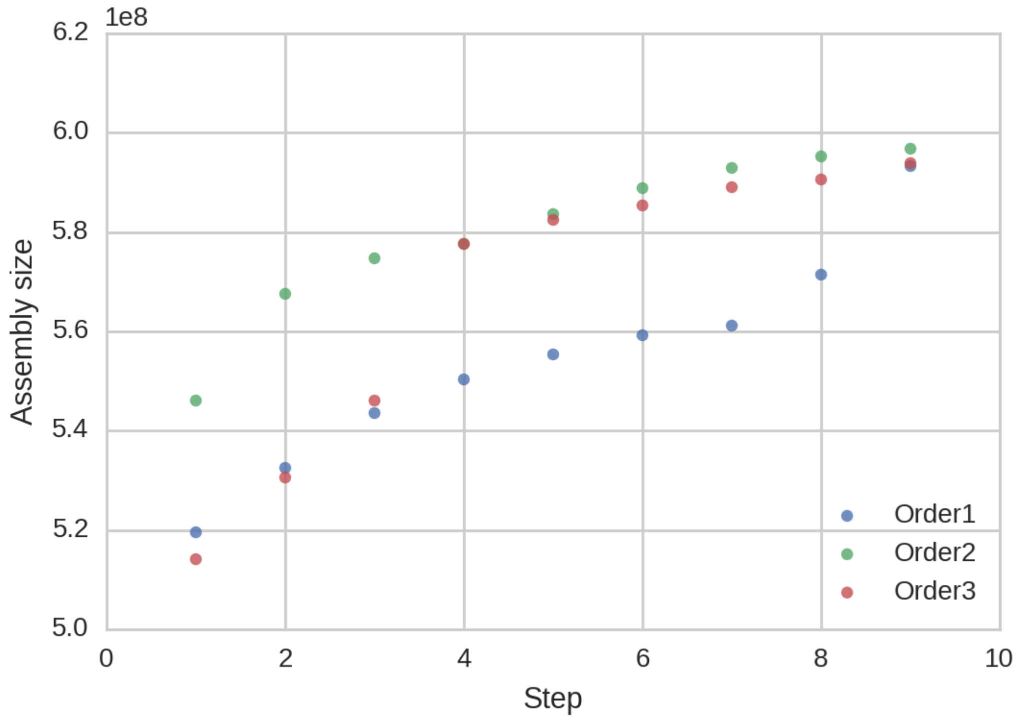

Supplementary Figure 9. Sizes of assemblies performed in three orders. Three assemblies were performed in different orders (two following inferred genetic relationships and one random):

1. Order1: Cabbage1, Cabbage2, Kale, Brussels Sprout, Kohlrabi, Cauliflower1, Cauliflower2, Broccoli, Macrocarpa
2. Order2: Macrocarpa, Broccoli, Cauliflower2, Cauliflower1, Kohlrabi, Brussels Sprout, Kale, Cabbage2, Cabbage1
3. Order3: Cauliflower1, Cabbage1, Broccoli, Macrocarpa, Kohlrabi, Cabbage2, Brussels Sprout, Cauliflower2, Kale

There was little difference in the overall assembly sizes, with the biggest assembly totaling 596 Mbp and the smallest assembly totaling 593 Mbp. The assembly chosen for the analysis was Order1. Using Order1 ensured that the maximum number of the genes incorporated into the pangenome originated from cultivated varieties not *B. macrocarpa*.

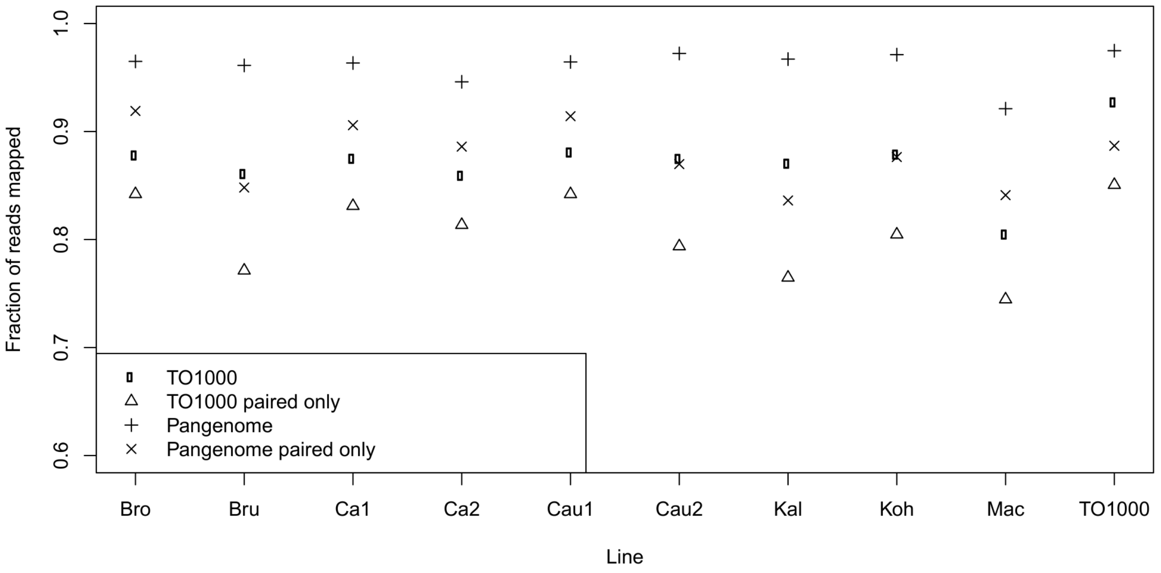

Supplementary Figure 10. Fraction of the reads mapped to the TO1000 portion of the pangenome only and the entire pangenome. The reads from all the lines were mapped to the pangenome. For each of the lines the percentage of reads mapping to the TO1000 portion of the pangenome only and the entire pangenome was calculated. The results suggest that the newly assembled contigs provide mapping space for the previously unmapped reads. Interestingly, an increased number of TO1000 reads mapping to the entire pangenome has been observed, possibly resulting from sequence missing from the original assembly. The number of the newly annotated genes which had TO1000 reads mapping to them was investigated. Only 121 such genes were identified, suggesting that almost entire TO1000 gene space has been captured in the original assembly. Reads mapping to mitochondrial, chloroplast and contamination sequences were excluded from the count.

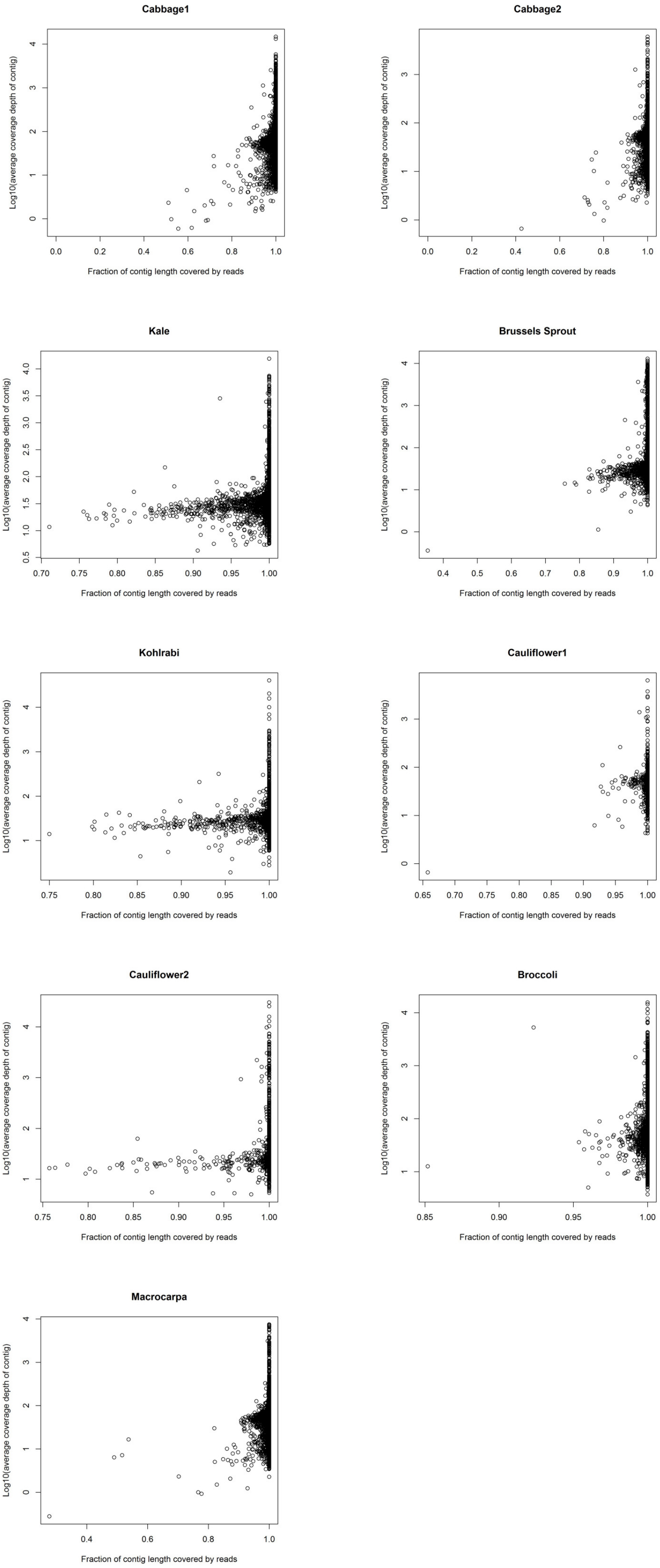
Cabbage1
Cabbage2
Kale
Brussels Sprout
Kohlrabi
Cauliflower1
Cauliflower2
Broccoli
Macrocarpa
Log10(average coverage depth of contig)
Fraction of contig length covered by reads

Supplementary Figure 11. Summary of coverage statistics while mapping reads from a given line to the contigs contributed by this line to the pangenome during iterative mapping and assembly.The horizontal (fraction of contig base pairs that has reads mapping) and vertical coverage (average coverage across contig) were calculated for all the contigs. The mappings were processed separately, for example while calculating coverage across contigs which stemmed from the assembly of unmapped reads from Cabbage1, only Cabbage1 reads were used in the calculation.

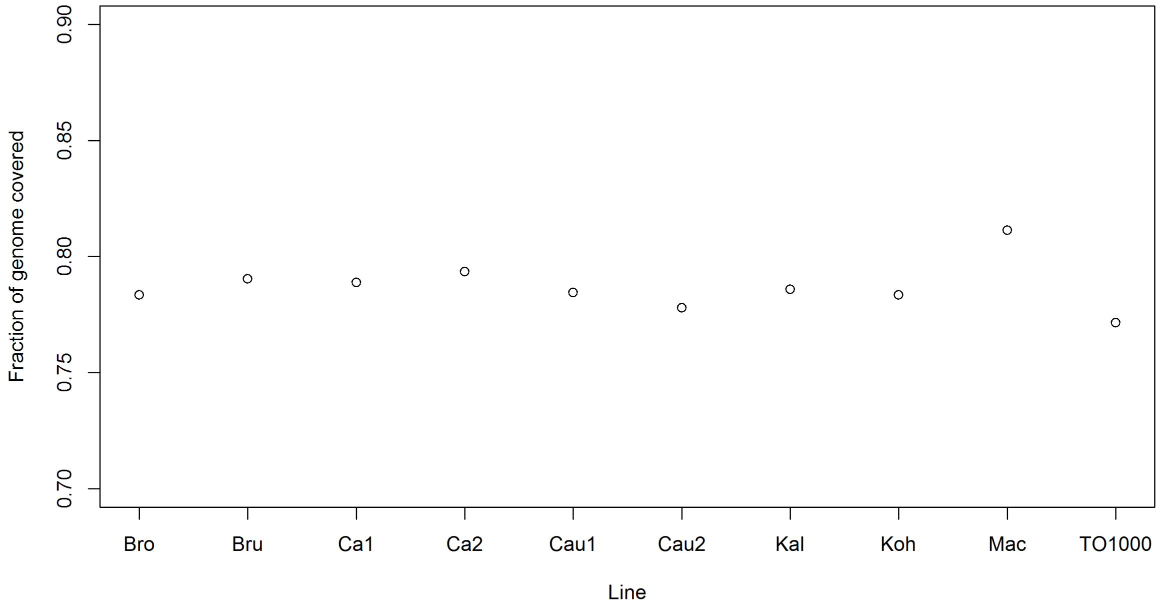

Supplementary Figure 12. The fraction of the pangenome which had reads mapping (coverage >=1) for each of the lines.

Supplementary Table 1. Sequence data used.

Name	Type	Alias	Technology	Insert size	Total # reads	Read lengths	By library Gbp	Total Gbp	Coverage
Early Big	Broccoli	Broccoli	Illumina PE	300	521,550,828	101	52.7	52.7	81
Badger Inbred 16	Cabbage	Cabbage1	Illumina PE	300	558,662,780	100	55.9	55.9	85.9
HRIGRU009617 DH3	Cabbage	Cabbage2	Illumina PE	300	491,221,854	100	49.1	49.1	75.6
BOL909	Cauliflower	Cauliflower1	Illumina PE	300	454,804,780	100	45.5	45.5	70
CA25 (Nedcha DH line)	Cauliflower	Cauliflower2	Illumina PE	300	237,618,810	100	2.7	27.5	42.2
				360		126	17.6		
				500		101	7.3		
AC498 (Gower DH line)	Brussels sprout	Brussels sprout	Illumina PE	300	278,524,304	101	8	30.8	47.4
				360		126	13.9		
				500		100	8.9		
ARS_18 (Arsis DH)	Kale	Kale	Illumina PE	300	260,951,324	101	8.7	29.2	44.9
				380		126	14.3		
				500		101	6.2		
HRIGRU011183 DH1	Kohlrabi	Kohlrabi	Illumina PE	300	250,947,876	101	7.3	28.2	43.5
				390		126	14.6		
				500		101	6.4		
TO1000 DH3	Chinese Kale (Rapid cycler)	Chinese kale/TO1000	Illumina PE	273	500,475,660	101	26.9	50.5	77.8
				418		101	23.7		
B. macrocarpa	B. macrocarpa	Macrocarpa	Illumina PE	300	506,114,122	100	50.6	50.6	77.9

Supplementary Table 2. Assembly statistics summary.

Line	Total length	# contigs	Longest contig	N50
Cabbage1	29,885,697	23,011	17,366	1,915
Cabbage2	12,302,363	11,790	16,776	1,457
Kale	10,925,979	12,001	17,981	1,338
Brussels sprout	6,618,538	7,745	17,768	1,228
Kohlrabi	4,941,752	6,172	12,242	1,078
Cauliflower1	3,855,815	4,526	19,572	1,069
Cauliflower2	1,946,083	3,250	12,161	596
Broccoli	6,343,073	10,708	11,014	568
Macrocarpa	21,749,321	21,617	15,153	1,370
Total newly assembled	98,568,621	100,820		
Total newly assembled alignable to the reference (93.03% identity)	4,577,809 (4.6%)			
TO1000	488,622,507			
Total	587,191,128			

Supplementary Table 3. Number of genes annotated and used in the analysis. The genome assembly of *B. oleracea* var TO1000 contained 59,225 gene models. Of those 57,467 were annotated on chromosomes and contigs >=1,000 bp. Gene models located on contigs smaller than 1000 bp were not used in the analysis as those were likely to represent partial gene models (mean length of B. oleracea gene > 1000 bp) and represented less reliable targets for PAV discovery. Additionally, 3,002 genes contained TE-related domains and were discarded from analysis. Finally, 9 genes had no TO1000 reads mapped to them and were also discarded. This resulted in 54,457 TO1000 gene models used. The annotation of newly assembled contigs resulted in 7,078 genes annotated on contigs >= 1,000 bp. Among those, 156 contained TE-related domains and were discarded from the analysis. This resulted in the final set of 61,379 genes used in the analysis.

	# of gene models	# of gene models used in the analysis	# of complete gene models used in the analysis
TO1000	59,225	54,457	51,248
Newly assembled contigs	7,078	6,922	3,945
Pangenome	66,303	61,379	55,193

Supplementary Table 4. Core and variable gene size statistics.

	Gene length (mean:median)	Coding sequence length (mean:median)	# of exons per gene (mean:median)
Core genes	1,841:1,449	1,093.5:894	4.8:3
Variable genes	1,083.9:840	635.5:468	3.5:3
Variable genes TO1000 only	1,148.9:810	683.5:483	3.1:2
Variable genes newly assembled contigs only	1,040:854	603:459	3.7:3

Supplementary Table 5. Number of SNPs discovered.

Chromosome	Chromosome size	# SNPs	SNP density (per Kbp)
C1	43,764,888	403,770	9.225889028
C2	52,886,895	511,743	9.676177813
C3	64,984,695	643,591	9.903731948
C4	53,719,093	503,296	9.369033837
C5	46,902,585	410,039	8.742353966
C6	39,822,476	369,717	9.284128892
C7	48,366,697	465,284	9.619925049
C8	41,758,685	398,058	9.532340398
C9	54,679,868	508,331	9.296492815
TO1000 unplaced scaffolds	41,736,625	198,884	4.765215204
Cabbage1	29,885,697	201,102	6.729038309
Cabbage2	12,302,363	60,281	4.89995296
Kale	10,925,979	53,368	4.884505086
Brussels sprout	6,618,538	25,582	3.865204068
Kohlrabi	4,941,752	16,315	3.301460697
Cauliflower1	3,855,815	13,400	3.475270468
Cauliflower2	1,946,083	4,909	2.522502894
Broccoli	6,343,073	17,579	2.77136965
Macrocarpa	21,749,321	9,832	0.45206009
Total	587,191,128	4,815,081	8.20019372

Supplementary Table 6. Significantly enriched GO terms among the variable genes.

GO ID	Term	Annotated	Significant	Expected	P value
GO:0006182	cGMP biosynthetic process	67	31	10.82	7.30E-09
GO:0009816	defense response to bacterium, incompati...	581	146	93.82	1.60E-08
GO:0051762	sesquiterpene biosynthetic process	36	20	5.81	7.30E-08
GO:0002237	response to molecule of bacterial origin	787	178	127.09	1.20E-06
GO:0010204	defense response signaling pathway, resi...	144	45	23.25	5.00E-06
GO:0009992	cellular water homeostasis	95	33	15.34	7.50E-06
GO:0010045	response to nickel cation	80	29	12.92	1.00E-05
GO:0046741	transport of virus in host, tissue to ti...	70	25	11.3	5.40E-05
GO:0006171	cAMP biosynthetic process	75	26	12.11	6.90E-05
GO:0000966	RNA 5'-end processing	71	25	11.47	7.10E-05
GO:0009870	defense response signaling pathway, resi...	152	43	24.55	0.00011
GO:0002230	positive regulation of defense response ...	54	20	8.72	0.00017
GO:0042742	defense response to bacterium	4862	913	785.14	0.00022
GO:0035304	regulation of protein dephosphorylation	1098	221	177.31	0.00024
GO:0009615	response to virus	1369	279	221.07	0.00027
GO:0006310	DNA recombination	948	193	153.09	0.00031
GO:0031146	SCF-dependent proteasomal ubiquitin-depe...	154	41	24.87	0.00063
GO:0007169	transmembrane receptor protein tyrosine ...	314	73	50.71	0.00067
GO:0046713	borate transport	56	19	9.04	0.00085

Supplementary Table 7. Functional annotation of orthologous gene clusters enriched in variable genes.

Cluster #	Functional annotation
cluster1001	DNA binding
cluster1006	Disease resistance protein (TIR-NBS-LRR class) family
cluster1011	Receptor like protein 6
cluster1015	DNAse I-like superfamily protein
cluster1016	Leucine-rich repeat transmembrane protein kinase protein
cluster1018	Domain of unknown function (DUF1985)
cluster1025	Nucleic acid-binding, OB-fold-like protein
cluster1031	Cysteine-type peptidases
cluster1032	Zinc ion binding;nucleic acid binding
cluster1033	Nucleic acid-binding, OB-fold-like protein
cluster1035	RPS5-like 1
cluster1038	Disease resistance protein (TIR-NBS-LRR class) family
cluster1072	N/A
cluster1122	Disease resistance protein (TIR-NBS-LRR class) family
cluster1143	N/A
cluster1449	F-box family protein
cluster1493	Polynucleotidyl transferase, ribonuclease H-like superfamily protein
cluster1585	N/A
cluster1919	F-box and associated interaction domains-containing protein

Supplementary Table 8. Summary of NB-ARC genes discovered.

Domains present	Count
LRR NB-ARC	97
LRR NB-ARC CC	25
LRR NB-ARC TIR	132
NB-ARC	114
NB-ARC CC	30
NB-ARC TIR	40
NB-ARC TIR CC	1
Total	439

Supplementary Table 9. Unique Macrocarpa genes potentially involved in defense response, response to cold, salt stress and water deprivation.

Function	Genes
defence response	BOLEPAN_00005801,BOLEPAN_00005802,BOLEPAN_00005803,BOLEPAN_00005806,BOLEPAN_00005813,BOLEPAN_00005816,BOLEPAN_00005818,BOLEPAN_00005841,BOLEPAN_00005860,BOLEPAN_00005868,BOLEPAN_00005877,BOLEPAN_00005892,BOLEPAN_00005956,BOLEPAN_00005967,BOLEPAN_00005985,BOLEPAN_00005996,

	BOLEPAN_00006003,BOLEPAN_00006014,BOLEPAN_00006018,BOLEPAN_00006025,BOLEPAN_00006041,BOLEPAN_00006042,BOLEPAN_00006062,BOLEPAN_00006073,BOLEPAN_00006094,BOLEPAN_00006107,BOLEPAN_00006111,BOLEPAN_00006153,BOLEPAN_00006154,BOLEPAN_00006250,BOLEPAN_00006291,BOLEPAN_00006296,BOLEPAN_00006297,BOLEPAN_00006306,BOLEPAN_00006324,BOLEPAN_00006338,BOLEPAN_00006339,BOLEPAN_00006341,BOLEPAN_00006367,BOLEPAN_00006369,BOLEPAN_00006437,BOLEPAN_00006450,BOLEPAN_00006451,BOLEPAN_00006461,BOLEPAN_00006470,BOLEPAN_00006471,BOLEPAN_00006472,BOLEPAN_00006473,BOLEPAN_00006531,BOLEPAN_00006620,BOLEPAN_00006622,BOLEPAN_00006627,BOLEPAN_00006641,BOLEPAN_00006726,BOLEPAN_00006740,BOLEPAN_00006770,BOLEPAN_00006771,BOLEPAN_00006787,BOLEPAN_00006792,BOLEPAN_00006832,BOLEPAN_00006834,BOLEPAN_00006842,BOLEPAN_00006857,BOLEPAN_00006874,BOLEPAN_00006887,BOLEPAN_00006897,BOLEPAN_00006915,BOLEPAN_00006921,BOLEPAN_00006931,BOLEPAN_00006954,BOLEPAN_00006959,BOLEPAN_00006961,BOLEPAN_00006978,BOLEPAN_00007008,BOLEPAN_00007024,BOLEPAN_00007071,BOLEPAN_00007072
response to cold	BOLEPAN_00005865,BOLEPAN_00005985,BOLEPAN_00006014,BOLEPAN_00006041,BOLEPAN_00006042,BOLEPAN_00006146,BOLEPAN_00006231,BOLEPAN_00006514,BOLEPAN_00006618,BOLEPAN_00006802,BOLEPAN_00006931
response to salt stress	BOLEPAN_00005848,BOLEPAN_00005877,BOLEPAN_00005899,BOLEPAN_00005985,BOLEPAN_00006002,BOLEPAN_00006003,BOLEPAN_00006085,BOLEPAN_00006133,BOLEPAN_00006278,BOLEPAN_00006392,BOLEPAN_00006399,BOLEPAN_00006432,BOLEPAN_00006509,BOLEPAN_00006514,BOLEPAN_00006587,BOLEPAN_00006609,BOLEPAN_00006733,BOLEPAN_00006802,BOLEPAN_00006874,BOLEPAN_00006881,BOLEPAN_00006894,BOLEPAN_00006954,BOLEPAN_00006981
response to water deprivation	BOLEPAN_00005832,BOLEPAN_00006121,BOLEPAN_00006514,BOLEPAN_00006531,BOLEPAN_00006640,BOLEPAN_00006802,BOLEPAN_00006881,BOLEPAN_00006894,BOLEPAN_00006895,BOLEPAN_00006896,BOLEPAN_00006974,BOLEPAN_00007052

Supplementary Table 10. Variable auxin related genes.

Gene	*A. thaliana* ortholog	*A. thaliana* protein product	Complete/Partial gene model
Bo1g008000	AT5G51470	Auxin-responsive GH3 family protein	C
Bo2g155590	AT5G27030	TPR3	C
Bo3g054650	AT3G53250	SAUR57	C
Bo8g109480	AT5G51470	Auxin-responsive GH3 family protein	C
Bo9g018860	AT5G65670	IAA9	C
Bo9g117680	AT1G48660	Auxin-responsive GH3 family protein	C
BOLEPAN_00000923	AT2G33230	YUCCA 7	C
BOLEPAN_00004584	AT5G51470	Auxin-responsive GH3 family protein	P
BOLEPAN_00004649	AT1G48660	Auxin-responsive GH3 family protein	C
BOLEPAN_00006139	AT4G32280	IAA29	C

Supplementary Table 11. Variable flowering time related genes.

Gene	*A. thaliana* ortholog	*A. thaliana* protein product	Complete/Partial gene model
Bo3g027760	AT2G34555	GA2OX3	C
Bo4g098320	AT3G63010	GID1B	C
Bo5g152700	AT3G02310	SEP2	C
Bo9g163730	AT5G15840	CO	C
BOLEPAN_00001559	AT5G65080	MAF5	C
BOLEPAN_00001774	AT2G38880	NUCLEAR FACTOR Y, SUBUNIT B1	P
BOLEPAN_00002402	AT5G10625	FPF1-like	C
BOLEPAN_00002580	AT1G79460	GA2	P
BOLEPAN_00002655	AT2G37678	FHY1	C
BOLEPAN_00002684	AT5G10140	FLC	C
BOLEPAN_00004088	AT1G18450	ARP4	P
BOLEPAN_00004443	AT5G25900	GA3	C
BOLEPAN_00005424	AT5G10140	FLC	P

BOLEPAN_000 06568	AT3G57300	INO80	P

Supplementary Table 12. Variable glucosinolate biosynthesis related genes.

Gene	*A. thaliana* ortholog	*A. thaliana* protein product	Complete/Partial gene model
Bo2g006900	AT5G05260	CYP79A2	C
Bo2g006910	AT5G05260	CYP79A2	C
Bo2g102190	AT4G03050	AOP3 (AOP2)	C
Bo3g036900	AT2G43820	UGT74F2	C
Bo5g125020	AT3G16400	NSP1	P
Bo9g006240	AT4G03050	AOP3 (AOP2)	C
BOLEPAN_00002 417	AT2G20610	SUR1	C
BOLEPAN_00006 883	AT1G74090	SOT18	C

Supplementary Table 13. Variable ascorbate biosynthesis related genes.

Gene	*A. thaliana* ortholog	*A. thaliana* protein product	Complete/Partial gene model
Bo7g116310	AT4G33670	L-galactose dehydrogenase	P
BOLEPAN_00000 815	AT4G33670	L-galactose dehydrogenase	C

Supplementary Table 14. PCR validation of a subset of genes (PRESENT – presence of gene determined based on read mapping, X - presence of gene determined by PCR results (amplification product present)).

Primer no.	Gene	Cabbage1	Kale	Cauliflower2	Kohlrabi	Brussels	Does PCR result agree?
	Bo00534s040	PRESENT			PRESENT		
1		X			X		AGREES
2		X			X		AGREES
3		X			X		AGREES
	Bo3g007000	PRESENT			PRESENT	PRESENT	
4		X			X	X	AGREES
	Bo4g167940	PRESENT		PRESENT		PRESENT	
5		X		X		X	AGREES
6		X		X		X	AGREES
	Bo4g173540		PRESENT		PRESENT		
7			X		X		AGREES
	Bo5g017760	PRESENT		PRESENT			
8		X		X			AGREES
	Bo5g141410	PRESENT	PRESENT				
9		X	X				AGREES
	Bo6g031440		PRESENT	PRESENT			
10			X	X			AGREES
	Bo6g077210			PRESENT	PRESENT	PRESENT	
11				X	X	X	AGREES
	Bo7g082590	PRESENT	PRESENT				
12		X	X				AGREES
	Bo8g081350	PRESENT		PRESENT		PRESENT	
13		X		X		X	AGREES
	Bo9g061240	PRESENT	PRESENT				
14		X	X				AGREES
15		X	X				AGREES

	BOLEPAN_0000156 2	PRESENT				PRESENT	
16		X				X	AGREES
	BOLEPAN_0000010 7	PRESENT			PRESENT	PRESENT	
17		X			X	X	AGREES
	BOLEPAN_0000077 1	PRESENT				PRESENT	
18		X				X	AGREES
19		X				X	AGREES
	BOLEPAN_0000236 1	PRESENT			PRESENT		
20		X			X		AGREES
	BOLEPAN_0000131 0	PRESENT	PRESENT/ ABSENT		PRESENT		
21		X			X		AGREES
	BOLEPAN_0000155 1	PRESENT	PRESENT			PRESENT	
22		X	X			X	AGREES
23		X	X			X	AGREES
	BOLEPAN_0000184 6	PRESENT			PRESENT	PRESENT	
24		X			X	X	AGREES
	BOLEPAN_0000226 1	PRESENT		PRESENT	PRESENT		
25		X		X	X		AGREES
	BOLEPAN_0000227 1	PRESENT				PRESENT	
26		X				X	AGREES
	BOLEPAN_0000227 8	PRESENT	PRESENT			PRESENT	

27		X	X			X	AGREES
	BOLEPAN_0000234<br>1	PRESENT	PRESENT				
28		X	X				AGREES
	BOLEPAN_0000244<br>4	PRESENT		PRESENT		PRESENT	
29		X		X		X	AGREES
	BOLEPAN_0000264<br>5	PRESENT			PRESENT		
30		X			X		AGREES
31		X			X		AGREES
	BOLEPAN_0000325<br>4		PRESENT	PRESENT	PRESENT		
32			X	X	X		AGREES
	BOLEPAN_0000417<br>3		PRESENT			PRESENT	
33			X			X	AGREES
	BOLEPAN_0000316<br>5				PRESENT	PRESENT	
34					X	X	AGREES
	BOLEPAN_0000396<br>8		PRESENT	PRESENT			
35			X	X			AGREES

Supplementary Table 15. Primers used for PCR validation presented in Supplementary Table 14.

Primer number	Name	Primer1	Primer2
	Bo00534s040		
1		TCCGTTGGAGAGGCAAGTAAC	TAAGGCGTGGAAAGATCCCAC
2		GAAGACCAAGATGACACGGGT	AATCTCCAAGGCCTCAAGAGC

3		GAAGTGTTCTTTGCTGGCTGG	GGCTTGTACCAGCGTAGTCTT
	Bo3g007000		
4		AAAGAAGCATCTGCAGCTCCT	AGCGTTTTCTTCCCATGTCCT
	Bo4g167940		
5		CGTTGAGGCTATTTGCGACTG	GAGAACGCCGTCGACTATGAT
6		TCACTCACATCACCGGTCAAG	ATGTCGGAGTGTCTTGCAGAG
	Bo4g173540		
7		GGTGAGCTTTTTGGAGCCATG	GCATCAATCTCACGGACATGC
	Bo5g017760		
8		CAATGACGGTGGAGACCTGAA	GAATGTCGTCGCAGTTAGCAC
	Bo5g141410		
9		TTTGTAGGAGCCACACAACCA	AGTTTAGCGAGTTTGCCGAGA
	Bo6g031440		
10		ACGGAGAAGTTCGAGGAACAC	AGCTTCAGTTTCTTCTCCCCG
	Bo6g077210		
11		AGGAATCTGGAATCGACGCTC	ACTGTGATACGTAGCTCCCCT
	Bo7g082590		
12		CCTTTGGCTTCACGGGAAAAG	GTCACATGCAGCATCGAACTC
	Bo8g081350		
13		GACGTGGCTTCTGTTTTGGAC	GAGCCGGTTTGTTGGTGATTC
	Bo9g061240		
14		AGAGCAGGAAAGAGTTGAGCC	CTTTTGCGCCGTAGTAATCCG
15		TCAGATTTTCCCACCGTCAGG	GATTCCAGAGAGGCACAACCA
	BOLEPAN_00001562		
16		GGCATCTCTGGAGGTTGTTGA	CTCGCACGGTGATTGAATTCC
	BOLEPAN_00000107		
17		CAAGGAATTTCACAGCACGCA	TTACACAGCCCGCTTGTAAGT
	BOLEPAN_00000771		
18		GGTGGACTGGTTTCATCTCGT	GTGTATTATTCCCCGCCGCTA

19		GCATATTGGATGTTCACCGCC	CGCACATGACATTGCTAGCTC
	BOLEPAN_00002361		
20		ATCCTCCCAAGTACCTGTCGA	GCCAAGCGACTGAAGAGGTAT
	BOLEPAN_00001310		
21		ACCAGGGCCTAGATGTTTTCG	GTTTCAGCAACGCCGATATCC
	BOLEPAN_00001551		
22		GGGCGATTGGCATGATCTTTC	TGGCAAGGATTATGGGGATGG
23		ATACCACCTTGCTTCCCATGG	CGCTTGGACAAGGATCCGATA
	BOLEPAN_00001846		
24		TACCGAGGGAGGCTCTGTTAA	TGCCGATAGATCACTGATGCC
	BOLEPAN_00002261		
25		TTCGGAGAACACGAAGGACAG	GGTCGTTCTCCTGCTTGGTAA
	BOLEPAN_00002271		
26		TTGCGCTTCCTAACAGTAGCA	AGAGTTCGCTGGACGTTCAAT
	BOLEPAN_00002278		
27		CCACGCGTAAAATAGCCACTG	TCCTGGGGTAAGCGTAAACAC
	BOLEPAN_00002341		
28		TTGATGACGATGGCTGGTGAA	ACGCAAGCATCGGTATATCGT
	BOLEPAN_00002444		
29		GTTACCGCTCACCCCCTAAAA	GCGATGCTCTTGACGATCAAC
	BOLEPAN_00002645		
30		AGTGGAACAAGGCTTGGGAAA	TTGCGTGGAAGCGTTTACAAG
31		GTCGGCATTTCCAGGAGAGAA	AAGACGGCAGAACTTCTCCAG
	BOLEPAN_00003254		
32		TTGCGGAGGTAGTGAGAGAGA	TCTTCGTCCTCCCATTCAAGC
	BOLEPAN_00004173		
33		GGAGCCCAAAACTTCGAGAGA	CCGACGTGGAAATTGTTGGAC
	BOLEPAN_00003165		
34		GAGGATGTTGGGATGCAGGAA	GAGTTTCCTTGAGCTCGGTGA

	BOLEPAN_00003968		
35		TTTGCTTCCTCGCCCTTACTT	TTGAGTGTGGTGATCGACGTT
